# Genome-Wide Identification and Comparative Analysis for OPT Family Genes in *Panax ginseng* and Eleven Flowering Plants

**DOI:** 10.3390/molecules24010015

**Published:** 2018-12-20

**Authors:** He Su, Yang Chu, Junqi Bai, Lu Gong, Juan Huang, Wen Xu, Jing Zhang, Xiaohui Qiu, Jiang Xu, Zhihai Huang

**Affiliations:** 1 The Second Clinical College of Guangzhou University of Chinese Medicine, Guangzhou 510006, China; suhe@cau.edu.cn (H.S.); baijunqi@126.com (J.B.); gonglu0904@126.com (L.G.); juanhuangzi@126.com (J.H.); freexuwen@163.com (W.X.); ginniezj@163.com (J.Z.); qiuxiaohui@gzucm.edu.cn (X.Q.); 2 Institute of Chinese Materia Medica, China Academy of Chinese Medicinal Sciences, Bejing 100700, China; ychu@icmm.ac.cn

**Keywords:** *Panax ginseng*, oligopeptide transporter, flowering plant, phylogeny, transcription factor

## Abstract

Herb genomics and comparative genomics provide a global platform to explore the genetics and biology of herbs at the genome level. *Panax ginseng* C.A. Meyer is an important medicinal plant for a variety of bioactive chemical compounds of which the biosynthesis may involve transport of a wide range of substrates mediated by oligopeptide transporters (OPT). However, information about the OPT family in the plant kingdom is still limited. Only 17 and 18 OPT genes have been characterized for *Oryza sativa* and *Arabidopsis* *thaliana*, respectively. Additionally, few comprehensive studies incorporating the phylogeny, gene structure, paralogs evolution, expression profiling, and co-expression network between transcription factors and OPT genes have been reported for ginseng and other species. In the present study, we performed those analyses comprehensively with both online tools and standalone tools. As a result, we identified a total of 268 non-redundant OPT genes from 12 flowering plants of which 37 were from ginseng. These OPT genes were clustered into two distinct clades in which clade-specific motif compositions were considerably conservative. The distribution of OPT paralogs was indicative of segmental duplication and subsequent structural variation. Expression patterns based on two sources of RNA-Sequence datasets suggested that some OPT genes were expressed in both an organ-specific and tissue-specific manner and might be involved in the functional development of plants. Further co-expression analysis of OPT genes and transcription factors indicated 141 positive and 11 negative links, which shows potent regulators for OPT genes. Overall, the data obtained from our study contribute to a better understanding of the complexity of the OPT gene family in ginseng and other flowering plants. This genetic resource will help improve the interpretation on mechanisms of metabolism transportation and signal transduction during plant development for *Panax ginseng*.

## 1. Introduction

Peptide transportation is a widely observed phenomenon of translocating small peptides across a membrane in a carrier-mediated, energy-dependent manner [1]. Transported peptides are often hydrolyzed and the resulting amino acids are used as substrates for protein synthesis, sources of nitrogen and carbon [2,3], and signals for biological processes such as quorum sensing [4], yeast mating [5], and metal homeostasis regulation [6,7]. There are three distinct protein families related to peptide transportation. The ATP binding cassette (ABC, TC 3.A.1) transporter superfamily is the largest transporter gene family. The members are able to translocate a wide variety of substrates including amino acids, sugars, peptides, proteins, and a large number of hydrophobic compounds and metabolites across extra-cellular and intracellular membranes [8,9]. In contrast to the ABC family, the proton-dependent oligopeptide transporter (PTR, TC 2.A.17) family utilizes a proton gradient other than ATP hydrolysis for dipeptide and tripeptide translocation [10]. The members of PTR proteins have been found in all kingdoms of life except the Archaea [1,11]. PTR also participates in amino acid and nitrate transportation [12]. In addition to dipeptides and tripeptides that are translocated by PTR proteins, tetra-peptides, penta-peptides, and some longer oligopeptides are translocated by a novel protein family known as the oligopeptide transporter (OPT, TC 2.A.67) family [10].

The OPT family is a group of electrochemical potential-driven transporters that catalyze their solutes in an energy-dependent symport manner. CaOPT1 was first cloned from *Candida albicans* (Robin) Berkhout and functional verified in *Schizosaccharomyces pombe* (Lindner) and subsequently defined as OPT but not an ABC or PTR protein by Jeff Becker’s laboratory [13,14,15]. OPTs are suggested to play diverse roles in long-distance sulfur distribution, metal homeostasis, nitrogen mobilization, heavy metal sequestration by transporting glutathione, peptides, and meta-chelates [16]. Phylogenetically, the OPTs can be divided into Oligopeptide Transporter (PT) and Stripe-like (YSL) clades [16,17]. Genes in the YSL clade have been found in Archaea, eubacteria, fungi, and plants but not in animals, which function as metal chelate transporters [6,18,19] consisting of mugeneic acids (MA) or nicotianamine (NA) while genes in the PT clade have only been identified in plants and fungi mediating long-distance metal distribution, nitrogen mobilization, glutathione translocation, and heavy metal sequestration [16,20,21,22,23,24,25].

In plants, the OPTs may play important roles in plant growth and abiotic and biotic stress responses [13,26]. The OPT member *ZmYS1*, which was first cloned by Curie et al. [6] but re-defined by Yen et al. [27], was proven to mediate the import of Fe‒phytosiderophore complexes from soils and long-distance transport of iron‒NA complexes [13]. Studies of two *AtOPT3* T-DNA mutants indicated that *AtOPT3* is of importance in both embryo development and iron deficiency signal transduction [1,7]. In addition, *AtOPT3* is found to be expressed in the phloem and functions in long-distance shoot-to-root signaling for Fe/Zn/Mn status. A lack of *AtOPT3* in *Arabidopsis thaliana* (*Arabidopsis*) led to the over-accumulation of cadmium in seeds [23]. Glutathione (GSH) is an essential sulfur-containing tripeptide that performs various important roles in plant processes, including detoxification of xenobiotics, heavy metal transport and resistance, controlling redox status, and long-distance transport of organic sulfur [22]. GSH is a precursor for plants to use to produce phytochelatins (PCs), which is the polymerized form of GSH, by which heavy metals can be transported to a central vacuole for detoxification [28]. AtOPT4 and AtOPT6 from *Arabidopsis* [21,22] and BjGT1 [25] from *Brassica juncea* (*B. juncea*) are all capable of translocating Cd‒GSH conjuncts. Moreover, GSH also plays important roles in plant growth and development in response to abiotic and biotic stresses. 

The majority of members of OPT proteins seem to contain 16 TMSs including a few of which appear to have 17 TMSs. A homology-based analysis for each TMSs in the OPT family indicated that the 16-TMS proteins might have been generated by three sequential duplications from 2-TMS protein precursors. Additionally, gene fusion might be responsible for the 17-TMS proteins [17]. However, although the OPT proteins have been studied for more than two decades, the majority of studies still focus on model plants such as yeast, *Arabidopsis*, and *Oryza sativa* (rice) [1,21,29,30]. Thanks to the rapid development of whole-genome sequencing techniques, an exponential increase in genome information has provided us with great opportunities to identify more OPT genes in non-model plants and make comparisons among multiple species simultaneously. However, to the best of our knowledge, genome-wide identification of OPT proteins has only been conducted in *Ganoderma lucidum* [31], *Populus trichocarpa*, and *Vitis vinifera* [32]. *Panax ginseng*, which is a Traditional Chinese Medicine, has been used for several millennia and has become more and more popular around the world. It is the most commonly used medicinally species in the *Panax* genera in contrast to the other four species: *Panax quinquefolius, Panax vietnamensis, Panax japonicus*, and *Panax notoginseng* [33]. Since we finished the genome assembly for *Panax ginseng (P. ginseng*) in our previous report [34], the interest in characterizing OPT genes in *P. ginseng* and comparing it with other genome-assembly-available species has increased. 

Although more plant genomes have been mapped in the last decade, studies on the genome-wide identification and comparison of OPT genes among species are still limited. Information on the phylogeny, gene structure, expression patterns, and regulatory networks of OPT genes remains to be discovered. In the present study, we identified OPT genes from *P. ginseng* and 11 flowering plants with the purpose of uncovering the phylogenetic relationships and gene structures of OPT genes in flowering plants as well as investigating the expression profiles and regulators of OPT genes in *P. ginseng*. Our analysis, which combines these types of information, provides new insights into both the structural and functional roles of OPT genes in ginseng and serves as a valuable resource for further study of the roles OPT genes play in plant development and transport of secondary metabolism.

## 2. Results and Discussion

### 2.1. Identification of OPT Genes in P. ginseng and 11 Other Flowering Plants 

We identified the OPT genes for *P. ginseng* and other species with TransportTP by setting as reference organisms Oryza sativa and *Arabidopsis thaliana* [35]. As a result, a total of 364 OPT candidates were identified in our study (Table 1). Seventeen of the 18 identified genes from *Arabidopsis* were in accordance with the reviewed records deposited in Swiss-Prot (release 2018_10), and, although the other gene *At5g45450*, was not recorded in Swiss-Prot, it was regarded as an OPT gene recorded in GenBank. However, only 12 OPT genes were identified from rice, of which the accession numbers were not in accordance with those records deposited in Swiss-Prot. This might be due to a different version of the rice genome being used. Considering our genome assembly did not scale to a chromosome level, we conducted a manual curation of the 39 OPTs identified from *P. ginseng*. Thereafter, 37 OPT genes were kept for further analysis. In addition, we identified 54 and 26 OPT genes from poplar and grape in our study, respectively, while only 20 and 18 genes were identified by Cao et al. [32]. These results suggested that our identification of OPT genes was accurate and comprehensive.

Since we found some OPTs were highly redundant (similar to each other with 100% similarity) within species, we removed the redundant OPTs in order to reduce the subsequent calculation consumption using CD-HIT software [36] by setting the sequence identity threshold to 100%. Lastly, a total of 268 OPT genes were kept. Because a candidate OPT gene named “GSVIVT01007176001” identified from grape contained too many “X”s, we excluded it from further analyses. In order to generate robust results from subsequent studies, we replaced those predicted OPT genes from *Arabidopsis* and rice with reviewed OPT genes retrieved from Swiss-Prot. Furthermore, we introduced two other experimentally verified OPT genes from *B. juncea* and *Zea mays* (*BjGT1* and *Maize_YS_1* respectively [6,25]) into our study. Lastly, 278 OPT genes were used for further analysis (Supplementary File 1).

### 2.2. Protein Properties of OPT Genes for OPT Genes Identified in P. ginseng and 11 Other Flowering Plants

**Figure 1:**
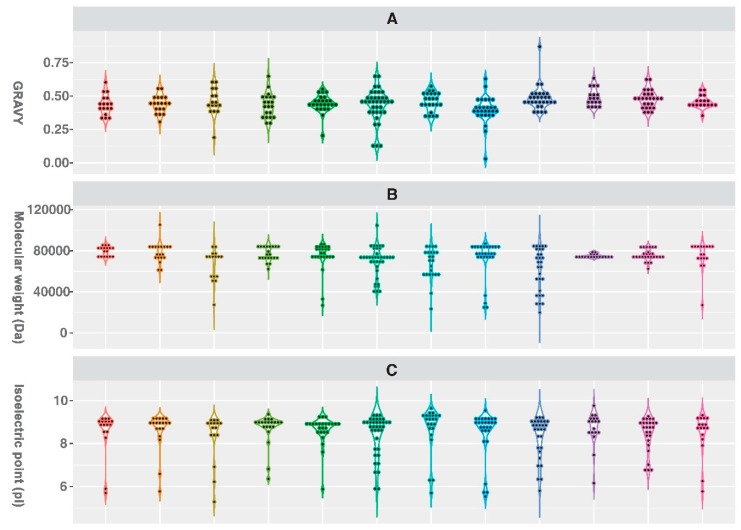

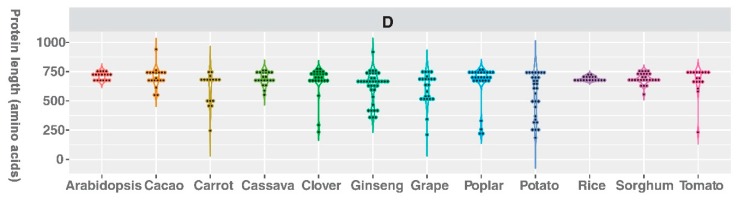
Protein properties for OPT genes identified from *P. ginseng* and 11 flowering plants. (**A**) Grand average of hydropathicity, GRAVY. (**B**) Molecular weight. (**C**), Isoelectric point, pI. (**D**) Number of amino acid residues.

**Figure 2:**
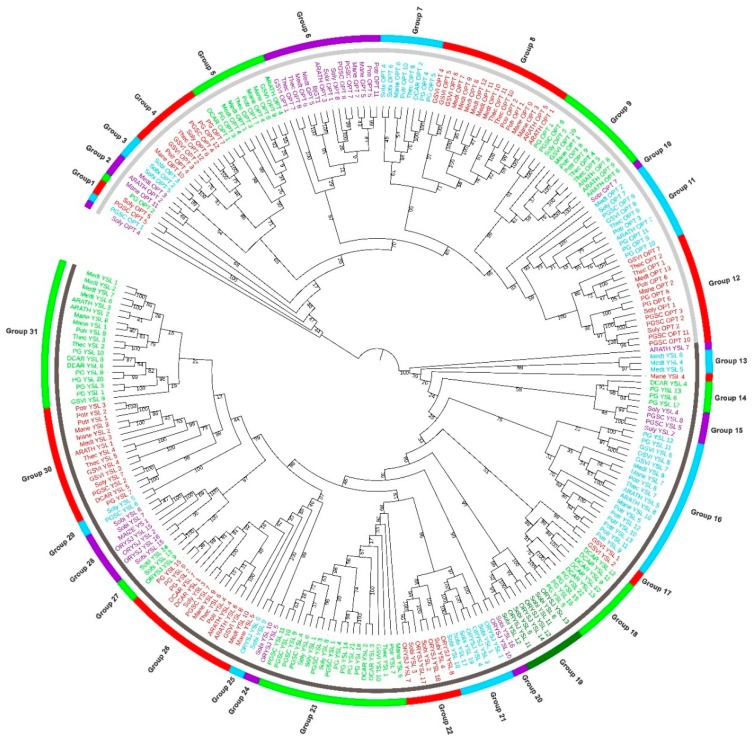
Phylogenetic relationships of OPT genes in *P. ginseng* and 11 other species. Tomato (Soly), Potato (PGSC), Cassava (Mane), *Arabidopsis* (ARATH), Clover (Medt), Poplar (Potr), Grape (GSVI), Cacao (Thec), Sorghum (Sobi), Carrot (DCAR), and Rice (ORYSJ). BjGT1 from *Brassica juncea* and Maize YS1 from *Zea Mays* (maize) are experimentally validated OPT proteins that were retrieved from GenBank database. Light gray in the inner circle indicates the PT clade. Dark gray refers to the YSL clade.

By examining the properties of OPT genes for each plant species, we found that the number of amino acid residues varied among species. Generally, the number of amino acid residues for OPT genes in *Arabidopsis*, rice, sorghum, and cassava ranged from 552 to 766, which is higher than the rest of those studied species (ranged from 184 to 941, as for *P. ginseng*). The number of residues ranged from 348 to 919 (Table S1, Figure 1D). The distribution of molecular weight for OPT genes was similar to the distribution pattern of residue numbers (Figure 1B). The grand average of hydropathicity (GRAVY) value is a measure of protein hydrophobicity [37]. Our results suggested that GRAVY for those OPT genes mainly ranged from 0.30 to 0.60 (Figure 1A). As OPT genes with the lowest and the highest GRAVY values (0.029 for Potri.017G150620.2.p and 0.87 for PGSC0003DMP400037534) were filtered out for lacking OPT-specific information for further phylogenetic analysis (Figure 2), we expanded the confident range of GRAVY values from 0.329–0.628 to 0.114–0.659 compared with the previous study [32]. In addition, the isoelectric point (pI) of the majority of OPT genes was around 9.0, which suggests that the electrochemical properties of OPT genes might be less varied in the plant kingdom (Figure 1C). 

Further analysis conducted with WOLF PSORT (http://woltpsort.org) enabled us to predict the probable protein localization for each candidate OPT identified in our study. It was found that all candidate OPTs were most likely to be located in the plasma and vacuolar membranes. The results were in accordance with a previous study [32]. Furthermore, 17 OPT genes were predicted to only be located in the plasma membrane. The remaining OPT genes were predicted to be not only in the plasma but also in at least one of the following: vacuole, chloroplast, cytoplasm, nucleus, mitochondria, Golgi apparatus, or endoplasmic reticulum (Table S1).

### 2.3. Phylogenetic Analyses, Classification, and Functional Relatedness of the OPT Genes Identified in P. ginseng and 11 Other Flowering Plants

To unravel the phylogenetic relationships of OPT genes in flowering plants, we conducted a phylogenetic analysis for those genes from 12 flowering plants. All OPT genes were clustered into two major distinct clades known as PT and YSL clade for which the results were consistent with those of previous reports [13,16,32]. However, what was different from previous studies was that the rice OPT genes in the PT clade were not included because no rice OPT genes in this clade were available in the Swiss-Prot database (release 2018_10). Therefore, only OPTs from the YSL clade were used in this phylogenetic analysis. Based on the bootstrap permutation test and the relationships of each OPT gene, we further classified the PT clade into 12 subgroups (Groups 1‒12) and the YSL clade into 19 subgroups (Groups 13‒31). Groups 23 and 31 included the largest number of members in the YSL clade (each with 19 members). Groups 9‒12 formed a highly confident larger group with a bootstrap value of 96% in the OPT clade and Groups 23 and 27‒30 formed another group in the YSL clade with a supporting value of 99%, which suggested that those members were likely to have evolved by recent gene duplication from a common ancestor. However, Soly_OPT_4 (Tomato), PGSC_OPT_1 (Potato), and PG_OPT_2 (Ginseng) failed to be grouped with any other PT genes due to a lack of supporting information by maximum likelihood analyses. In addition, ARTH_YSL_7 (*Arabidopsis*) and Mane_YSL_4 (Cassava) also failed to be grouped with any other genes in the YSL clade. Although Sobi_OPT_7 (Sorghum) seemed likely to stand alone, it was in fact grouped with Groups 11 and 12 with a bootstrap value of 93% (Figure 2). Furthermore, the motif structures of the genes described below also supported the group classifications (Figure S1). Moreover, ARATH_OPT_3 and BjGT3 (*Brassica juncea*), ARATH_OPT_1 and ARATH_OPT_5, ARATH_OPT_6 and ARATH_OPT_8 and ARATH_OPT_9, ARATH_YSL_5 and ARATH_YSL_8, ARATH_YSL_4 and ARATH_YSL_6, ORYSJ_YSL_7 (rice) and ORYSJ_YSL_17 were grouped together, with the phylogenetic relationships in accordance with previous study reports [16,30,32]. The consistency of our findings with previous findings indicated that our phylogenetic study was properly conducted and the results were reliable. However, it was interesting to find out that ARATH_YSL_7, which has been reported to be sub-grouped with ARATH_YSL_5 and ARATH_YSL_8 [30], failed to be grouped with any other OPT members in the YSL clade in our study.

Genes with the same functions were often closely related, as found in both a previous study [32] and our study. *BjGT1*, which is the first cloned and characterized OPT gene from *Brassica juncea*, was experimentally validated to be a glutathione transporter mediating cadmium absorption [25]. ARATH_OPT_3, which is another OPT gene that was cloned and characterized in *Arabidopsis*, was reported to be involved in the sensing and translocation of Cd (as well as Fe and Zn) [1,7,23]. These two functionally similar genes were clustered together in our study. In addition, Mazie_YS_1, the first experimentally validated OPT gene responsible for transport of Fe(III)-phytosiderophore chelates, was clustered together with ORYSJ_YSL_15 and ORYSJ_YSL_2 (Figure 2). ORYSJ_YSL_15 has been suggested to be responsible for iron uptake from rhizosphere and for phloem transport of iron by transporting Fe(III)-phytosiderophore chelates while ORYSJ_YSL_2 has been suggested to be responsible for phloem transport of iron by transporting Fe(III)-nicotianamine chelates [38,39]. Furthermore, ARATH_YSL_2 and ARATH_YSL_3 clustered together in Group 31 were both reported to be involved in transport of nicotianamine-chelated metals in the vasculature [40,41]. These results supported the idea that genes with the same functions were closely related. Based on the hypothesis, it would be interesting to test if PG_YSL_1 is involved in iron-transportation since it was clustered together with ARATH_YSL_1 that was found to be involved in transport of iron-nicotianamine chelates [41,42]. Similarly, it would be interesting to test if PG_OPT_1 is akin to ARATH_OPT_6, which was reported to be involved in the transport of glutathione derivatives and metal complexes [21,43,44] and to test whether PG_OPT_9,10 and PG_OPT_11 are involved in increasing plant sensitivity to Cd like ARATH_OPT_7 functions [43].

**Figure 3:**
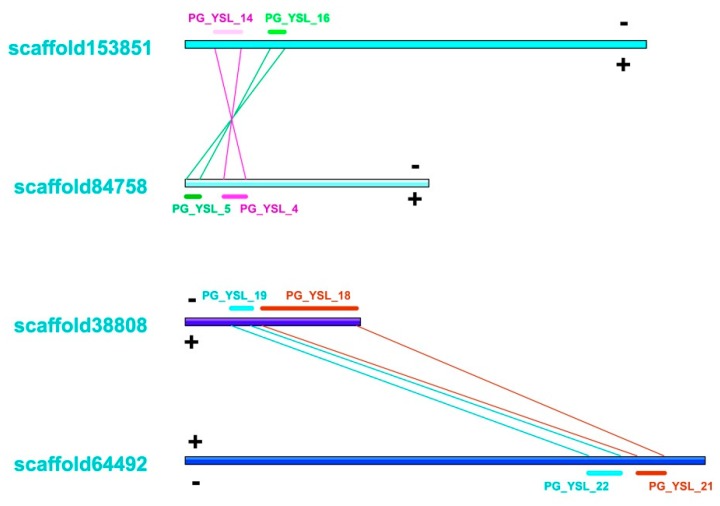
Chromosome locations for two special types of clusters of paralogs blocks. We used the ginseng genome version1 finished by Xu et al. [34] in this study. The scaffold refers to the DNA sequences in the ginseng genome that were generated by bridging non-gapped contigs (assembled with short gun sequencing reads) with mate-pair sequencing reads. A scaffold is equivalent to a chromosome segment.

We identified a total of 45 pairs of paralogs from the phylogenetic analyses (Table S2), which accounted for 11.1% to 70.6% of all OPT candidates in each studied species and shared similar structures within each group (Figure S1). We found that some OPT genes in ginseng were tandemly clustered on the same scaffold (Table S3) and those genes were location-related. For example, PG_OPT_10 and PG_OPT_11 were neighbor paralogs with 1122 bp in between. These genes might be formed by tandemly segmental duplication. PG_YSL_8 and PG_YSL_10 constitute a special tandemly clustered paralogs with a 3214 bp-long shared region. This paralogs pair might be generated by a crossover of chromosome after whole-genome duplication or by gene fusion. It would be interesting to test whether this OPT cluster was functional in further studies. In addition, PG_YSL_4-PG_YSL_5 and PG_YSL_14-PG_YSL_16 formed a special type of gene cluster block in which PG_YSL_4-PG_YSL_14 and PG_YSL_5-PG_YSL_16 were identified as paralogs oriented in the same direction. PG_YSL_18-PG_YSL_19 and PG_YSL_21-PG_YSL_22 constituted another special type of block, in which PG_YSL_18-PG_YSL_21 and PG_YSL_19-PG_YSL_22 were paralogs oriented in opposite directions (Figure 3). From this section of the study, we speculated that both types of cluster blocks were generated from segmental duplication or whole-genome duplication. Since *P. ginseng* is a tetraploid plant, we prefer to believe that genes from those blocks were more likely to be generated by whole-genome duplication. The paralogs blocks arranged in the opposite direction were likely to be generated by subsequent segmental inversion of the chromosome after segmental duplication.

*Ks* (synonymous substitution rate) is a widely accepted concept for gene duplication time estimation. In general, the lower *Ks* is, the more recently gene duplication occurred [32]. Since a codon-based alignment of PG_YSL_6 and PG_YSL_17 failed to generate, calculation of *Ks* was excluded from this study (Table S2). Aligned sequences were nearly identical after removing gaps from Potr_YSL_2/Potr_YSL_3 (poplar), Thec_OPT_7/Thec_OPT_8 (cacao), and Thec_YSL_4/Thec_YSL_5. The estimation of *Ks* for these paralogs also failed. Additionally, *Ks* values for PG_OPT_9/PG_OPT_10 and Sobi_YSL_13/Sobi_YSL_14 were estimated as 0, suggested that they were generated by a very recent duplication event. It was interesting to find that gene duplication of OPT paralogs occurred more recently in the YSL clade than in the PT clade in *P. ginseng*. The phenomenon was similar to grape and clover but contrary to cacao, cassava, and *Arabidopsis*. The duplication event for paralogs occurred more recently in ginseng, potato, poplar, cacao, grape, clover, and sorghum (about 0 to 5 MYA) than in carrot, cassava, *Arabidopsis*, and rice, which indicates that ginseng and other species or their common ancestor might have suffered a high level of gene loss during evolution because of the lack of an older duplication event such as 94.2 MYA for ARATH_OPT_6/ARATH_OPT_9 [45].

### 2.4. Conserved Domains and Motif Analysis for OPT Genes Identified in P. ginseng and 11 Other Flowering Plants

**Figure 4:**
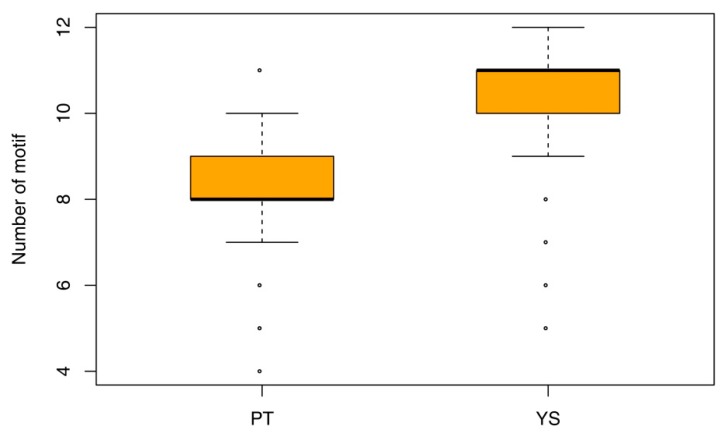
Number of motifs in OPT genes from PT and YS clades. These static results were calculated with xml output of MEME analysis (Supplementary File 5) by our custom R scripts. The boxplot was generated by the built-in function “boxplot” in R.

By searching against the Conserved Domain Database (CDD) [46] with 278 OPT genes, all genes were annotated as OPT genes. However, only 267 were predicted to have specific domains, wherein all the 258 OPT genes used in the phylogeny analysis were covered. Because domain analysis could not provide information about smaller individual motifs and more divergent patterns, we conducted a study of motif analysis with MEME software (Supplementary File 2). As a result, 30 distinct motifs were identified in these genes. Detailed information of those motifs is presented in Supplementary File 3. It is interesting that the motif composition of OPT members in the PT clade is distinct from that in the YSL clade (Figure S1), which was in accordance with the conclusions generated by phylogenetic analysis. In addition, the number of motifs of OPT genes from the PT clade (ranging from 4 to 11, with a median value of 8) was distinct from that of the YSL clade (ranging from 5 to 12, with a median value of 11), which suggests the clade-specific structure of each OPT gene (Figure 4). Furthermore, we found nine motifs (Motif_1,3,6,13,14,19,23,15,29) unique to the PT clade and 10 motifs (Motif_7,8,12,16,17,21,22,24,26,30) unique to the YSL clade, respectively. Six motifs (Motif_10,14,19,23,102,106) were frequently shared by PT clade members (94.4%, 94.4%, 86.9%, 91.6%, 95.3%, and 99.1%, respectively) and 10 motifs (Motif_2,8,9,15,16,18,21,22,26,28) were frequently shared by the YSL clade members (92.0%, 88.7%, 71.3%, 38.7%, 92.0%, 92.0%, 92.7%, 81.3%, 84.7%, 97.3%, and 94.0%, respectively) (Table S4). Those findings might give us new insights into how OPT genes evolved since being separated from their common ancestor and how they functionally diverged during the subsequent evolution process. 

### 2.5. Profiling of Expression Patterns for OPT Genes Identified in P. ginseng

**Figure 5:**
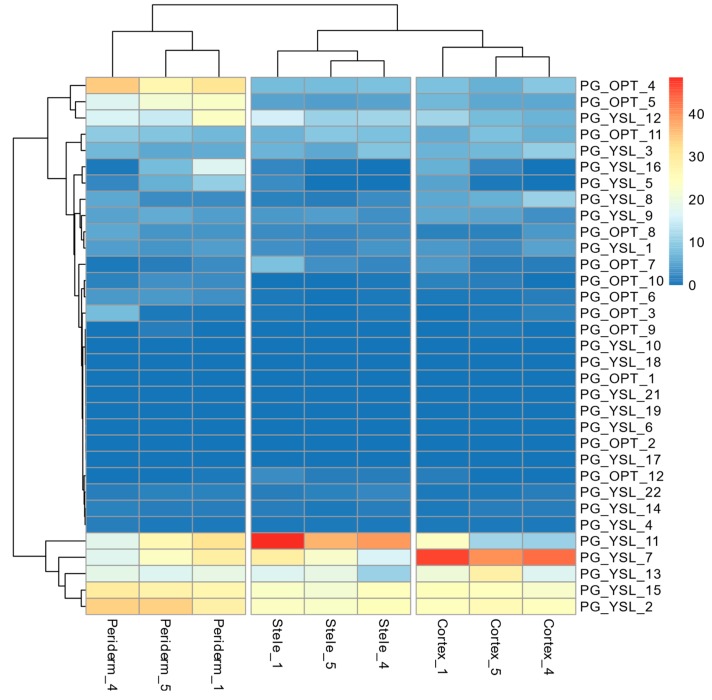
Expression of OPT genes of different tissues in ginseng root. Gene expression of OPT genes was calculated with RNA-Seq data generated by our previous study on *P. ginseng* root. The hierarchical clustered heat map was plotted with ‘pheatmap’ planted in R package named ‘pheatmap’.

In order to examine the expression patterns of the OPT genes in *P. ginseng*, we performed a comprehensive expression analysis by using two sets of RNA-Seq datasets: one from our previous study about *P. ginseng* root [47] and one from a public study about 18 kinds of tissues. In general, genes in the YSL clade were more highly expressed than genes in the PT clade except in the periderm (Figure 5). PG_YSL_2,13 and PG_YSL_15 were expressed evenly in the root with little difference among tissues, which suggests that they might be constitutive OPTs. OPT genes exhibited distinct tissue-specific expression manners. For example, PG_OPT_4,5 and PG_YSL_12 were more likely to be expressed highly in periderm than in the stele or cortex. PG_YSL_11 and PG_YSL_7 had the highest expression in the stele and cortex, respectively, while they were still expressed at a considerably high level in other tissues. The different expression patterns for those OPT genes indicated that a wide range of substrates might be transported in different parts of the plant root.

**Figure 6:**
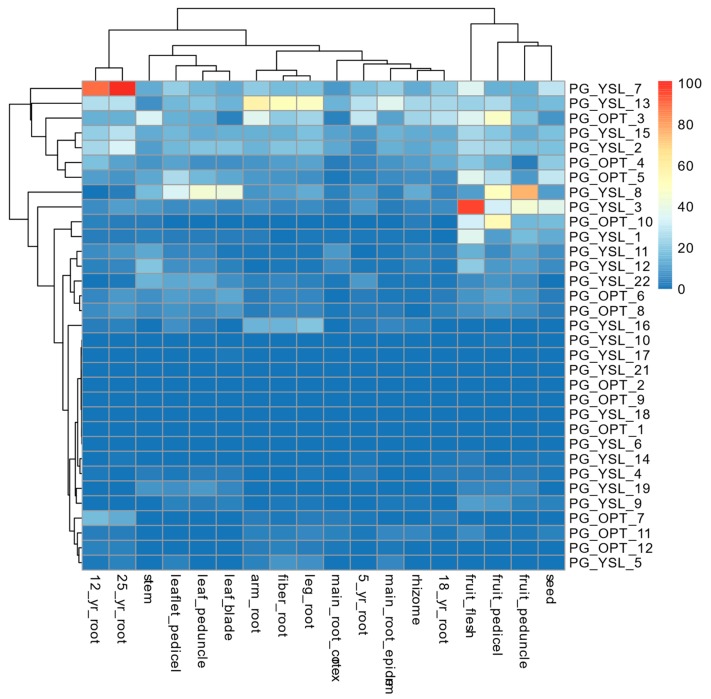
Expression of the OPT genes in 18 organs of ginseng. Gene expression of OPT genes was re-calculated with RNA-seq data from public research by taking our published genome as a reference.

Due to the nature of sink tissue of fruit and seeds in plants, the expression characteristics of OPT genes of these tissues are expected to share more common traits than those of other tissues. Based on the expression data, fruit flesh, fruit pedicel, fruit peduncle, and seeds were clustered together, which suggests that the similar expression pattern of those OPT genes might contribute to methods of metabolism relocation. The expression of PG_YSL_1,3 and PG_YSL_7 both peaked in fruit flesh compared with the fruit pedicel, fruit peduncle, and seed, which indicates that lateral transportation might be the most active transportation process during seed development. Additionally, the leaf blade, leaf pedicel, leaf peduncle, and stem, which are physically connected organs forming a complex vascular transportation system in plants, were clustered together by their similar expression pattern, wherein PG_YSL_8 was expressed at the highest level (except for the stem). Moreover, the arm root, fiber root, and leg root were clustered together, and PG_OPT_13 and PG_YSL_16 were highly expressed. It was interesting that PG_YSL_7 was highly expressed in 12-year-old and 25-year-old roots but minimally expressed in five-year-old and 18-year-old roots that were clustered with the main root cortex, the main root epiderm, and the rhizome. Taken together, the expression patterns found in our study and Wang’s [48] both suggested that OPT genes were expressed in tissue-specific and location-specific manners by which the transportation and distribution of oligopeptides and their conjugates with metals, signals, etc. were shaped in different ginseng tissues [41] (Figure 6).

Based on the phylogenetic analysis described above, PG_OPT_4 and PG_OPT_5 were grouped with ARATH_OPT_1 and ARATH_OPT_5, which were proven to be OPT transporters for penta-peptide (KLLLG) in an energy-dependent manner by yeast complementation assay [20,49]. High expression of those genes exclusively in the root suggested that the penta-peptide-related metabolism (metabolism substrates, signal molecules, etc.) transportation might be activated. PG_YSL_12 was identified as another periderm-specific expressed gene found in this study. It was expressed more highly in the stem and fruit flesh than in other tissues (Figure 6). PG_YSL_12 was clustered with ARATH_YSL_5 and ARATH_YSL_8 into one group, which indicates that it might be involved in the transport of nicotianamine-chelated metals (metals‒NA) just as ARATH_YSL_2 was in the transport of Fe‒NA across the plasma membrane in leaf cells, involving lateral movement of iron away from the xylem [40]. Furthermore, ARATH_YSL_8 might also be involved directly in iron uptake by leaf cells [13].

ARATH_YSL_1 and ARATH_YSL_3 were experimentally verified OPT proteins, which were found to be able to mediate Fe transportation to and from vascular tissues [41]. ARATH_YSL_3 was a sister branch to ARATH_YSL_2, which had been functionally confirmed to be expressed in both roots and shoots and to mediate transport of metal‒NA complexes [40], which indicates their functional similarity. ARATH_YSL_1 was clustered into another sister group to a larger group including ARATH_YSL_2 and ARATH_YSL_3, which suggests that members of these two larger group might share some functional similarities. MAIZE_YS_1 (*ZmYS1*) known as a proton-coupled symporter transports iron complexed by plant-derived Fe(III) chelators (phytosiderophores, PS) by scavenging from soil, termed Strategy II [13]. It formed another cluster in the YSL clade with some YSL genes from sorghum and rice with a bootstrap value of 97% (Figure 2). Considering the functional similarity of ARATH_YSL_1,2,3 and *ZmYS1*, OPT genes from groups 27 and 28 and groups 29‒31 were suggested to form two sister groups that might be involved in the transport of Fe. 

The OPT paralogs were more likely to be generated by segmental tandem duplication rather than transposition [32]. The expression pattern of those duplicated genes may differ if they suffered evolutionary divergence such as neofunctionalization. [50]. No similar expression patterns of duplicated paralogs were identified in the study about poplar and grape [32]. However, we detected five similarly expressed paralogs pairs in this study wherein PG_OPT_4-PG_OPT_5 had similar expression patterns both in our previous study and in Wang’s study. PG_YSL_4-PG_YSL_14 and PG_YSL_18-PG_YSL_21 were expressed similarly but with a very low expression level. PG_YSL_6-PG_YSL_8 and PG_YSL_11-PG_YSL_12 were reported to be expressed similarly in Wang’s report but not in ours. However, PG_YSL_5/PG_YSL_16 was similarly expressed in our study but not in Wang’s study. The similar expression patterns found in our study might be because of the relatively short time has been experienced in *Ginseng* paralogs compared with those paralogs in poplar and grape. On the other hand, the phenomenon that a majority of the identified paralogs in *Ginseng* did not have similar expression patterns, which indicates functional diversificationmight be a result of long-term evolution—adapting to changing environmental conditions after gene duplication. 

### 2.6. Analysis of Co-Expression Network between OPT Genes and Potent Transcription factOr for P. ginseng

The regulation of gene expression in all living cells is dominated by transcriptional initiation, which is regulated by transcription factors, ancillary transcription regulators, and chromatin regulators. Therefore, we conducted an analysis focusing on the co-expression between all transcriptionally modulated genes in the ginseng genome and all transcription factors in order to reveal regulators for OPT genes in *Ginseng*. PlantTFcat is a useful tool for identifying proteins with signature domains specific to 108 major transcription regulators families [51]. We assessed the *Ginseng* genome for identifying those proteins. A total of 5073 distinct genes in the *P. ginseng* genome have been predicted to be transcription factors wherein there are 5457 members (Supplementary File 4). Genes annotated as different transcription factors by PlantTFcat (such as PG39956, annotated as Znf-B, LisH, WD40-like, or PLATZ) were removed from further analysis. The expression values of transcription factors that were mapped by many genes (such as MYB-HB-like, mapped by 334 genes) were determined by the median values of those genes. Lastly, a total of 59 transcription factors and 13 OPT genes were used for network analysis. We used non-parametric Spearman’s rank-order correlation for our co-expression analysis due to its robustness for generating biologically relevant gene networks [52]. 

**Figure 7:**
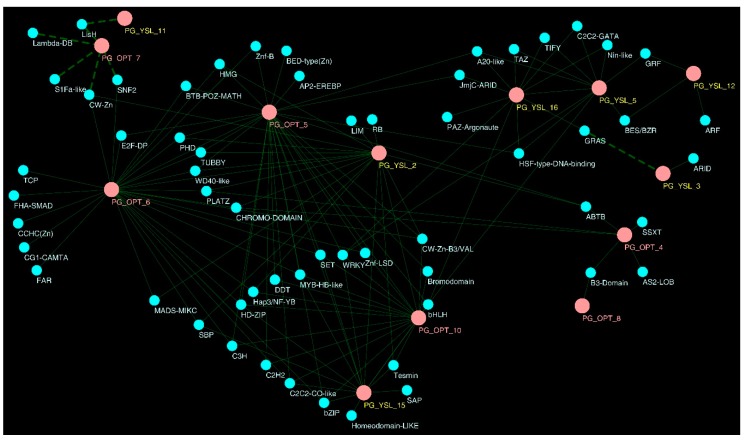
Regulatory gene networks involving transcription master genes and OPT genes. A stringent threshold (ρ ≥ |0.85|) was used and the visualization was produced in Cytoscape-3.6.1. Nodes for OPT genes are represented by pink circles (yellow labels represent OPT genes in the YSL clade. Pink labels represent OPT genes in PT clade). Nodes for blue circles represent transcriptional regulators. Positive interactions are indicated by green lines and negative interactions are indicated by green dashed lines.

The matrix of all correlation values for expression values between each pair of transcription factor and OPT gene from a set of nine biological samples is shown in Table S5**.** At a conservative threshold of ρ ≥ |0.85|, 141positive and 11 negative correlations involving 13 OPT genes were found (Table S6). The number of transcription factors correlated to an OPT gene ranged from 1 to 27, while it ranged only 1 to 5 for the number of OPT genes correlated with a transcription factor (Tables S7 and S8, Figure 7). For example, PG_OPT_5 and PG_OPT_6 positively correlated with 26 and 27 transcription factors, respectively, while *bHLH* and *WRKY* only correlated with five OPT genes. Our findings suggested that the initiation of transcription of OPT genes might be dominated by a complicated synergetic regulation system consisting of a number of transcription factors. Additionally, transcription factors might act as pleiotropic regulators participating in a variety of transcription regulations for OPT genes. On the other hand, because 19 out of 59 transcription factors were linked to only one OPT gene and two out of 13 OPT genes were linked to only one transcription factor, the results suggested that the transcription of some OPT genes was regulated by specific transcription factors and some transcription factors had specific target genes to regulate.

## 3. Materials and Methods 

### 3.1. Sequence Retrieval and Identification of OPT Genes

We identified OPT genes from *P. ginseng* and 11 other flowering plants by using TransportTP (http://bioinfo3.noble.org/transporter/ [35]). Proteome sequences from 11 flowering plants (*Arabidopsis* TAIR10, rice v7, tomato iTAG2.4, potato v4.03, carrot v2.0, *Manihot esculenta* v6.1, *Medicago truncatula* Mt4.0v1, Poplar v3.1, Grape Genoscope 12X, Cacao v1.1, Sorghum v3.1.1) were retrieved from the Phytozome database (V12.1) [53], wherein the assembly version is followed by each species name. Ginseng proteome sequences were retrieved from http://ginseng.vicp.io:23488/index.php/index/download.html. The proteome sequences for each species were then used for the identification of OPT genes by searching against TransporterTP, setting the E-value threshold to 0.1, and setting *Arabidopsis thaliana* and *Oryza sativa* as the reference organisms. 

Subcellular localization of those OPT proteins was predicted with WOLF-PSORT [54,55]. Isoelectric point (pI), molecular weight, and grand average hydropathicity (GRAVY) values were estimated with functions planted in the Peptides package (https://github.com/dosorio/Peptides/) for R. 

### 3.2. Phylogenetic Analysis for OPT Genes

Phylogenetic analysis of these OPT genes was conducted on their conserved domains identified by CDD (Conserved Domain Database, [56,57]) and planted in NCBI with default parameters (50369 PSSMs, e-value of 0.01, maximum number of hits 500). Multiple sequence alignments of those conserved OPT protein were performed with MAFFT v7.158b [58] and followed by manual comparison and refinement. Aligned regions that contain over 50% gaps or ambiguous sites were removed and sequences that contained gaps for more than 50% of the remaining sequence were deleted. Lastly, 258 OPT genes were left for further phylogenetic analysis. In order to select the best evolutionary model for phylogeny reconstruction, we used the function ‘modelTest’ planted in the R package named ‘phangorn’ [59] with the parameter ‘model’ set to ‘all’ and found ‘LG+G+F’ was the best model. A maximum likelihood method of phylogenetic analysis based on RAxML v 8.2.9 [60] software was conducted, with the parameter “--bootstop-perms” set to 1000, “-m” set to PROTGAMMALG, and the “-e” set to 0.001. After finishing the reconstruction of the phylogeny of OPT genes for these species, the topology was plotted by the online tool iTOL [61].

### 3.3. Estimation of Duplication Time for OPT Paralogs

Pairwise alignment of protein sequences of the OPT paralogs was aligned with MAFFT software, and codon-based pairwise alignment of nucleotide sequences were generated by using PAL2NAL [62]. The *Ka* and *Ks* values for paralogous genes were estimated by the program yn00 planted in the PAML package with default parameters [63]. Assuming a molecular clock, the synonymous substitution rates (*Ks*) of the paralogous genes could be regarded as a proxy for time estimation of the segmental duplication events. The approximation of date for duplication events was estimated with the following formula: T = *Ks/2*λ, where λ denotes clock-like rates of synonymous substitution. In this study, 1.5 × 10^−8^ substitutions/synonymous site/year was used for *Arabidopsis*, 6.5 × 10^−9^ for rice, sorghum, cassava, grape and cacao, 9.1 × 10^−9^ for poplar [32], 1.08 × 10^−8^ for clover, 6.68 × 10^−9^ for *P. ginseng*, 2.69 × 10^−9^ for potato, and 2.91 × 10^−9^ for carrot. λ for each species was deduced or collected from previous studies [64,65].

### 3.4. Analysis of Motif Composition for OPT Genes

Conserved motif analysis for OPT genes in the *P. ginseng* genome was conducted with MEME (http://meme.sdsc.edu). The OPT candidates were run locally with MEME with the following parameters: number of repetitions = any, maximum number of motifs = 30. The other parameters were kept as default values.

### 3.5. Profiling Expression of OPT Genes for P. ginseng

The gene expression of *P. ginseng* was profiled by RNA-Seq datasets from our previous study [47] and public research [48]. Those datasets could be retrieved from the SRA database by searching BioProject id PRJNA369187 and PRJNA302556. These raw datasets from the SRA database were first converted into FASTQ files by sratoolkit.2.8.0 [66] and then quality controlled by Trimmomatic-0.36 [67]. Lastly, reference-based gene expression of those biological samples was estimated with the HISAT2+StringTie pipeline [68]. FPKM values for each gene were used as gene expression levels. A hierarchical clustered heatmap for OPT genes was plotted with the pheatmap package [69], wherein “manhattan” distance was used for both row-based and column-based clustering. 

### 3.6. Identification of Regulatory Network between OPT Genes and Transcription Factors for P. ginseng 

The *P. ginseng* proteome sequence dataset was submitted to the PlantTFcat analysis tool (http://plantgrn.noble.org/PlantTFcat/ [51]) for the identification and classification of transcription factors, chromatin modifiers, and other transcriptional regulators into protein families. Genes that mapped to more than one transcription factors were removed from further analysis. In addition, median values of those genes referring to the same transcription factors were regarded as the transcription factors’ expression value. In this study, we used our previous RNA-Seq dataset for construction of the co-expression network between OPT genes and transcription factors. FPKMs of all genes including OPT genes and transcription factors were combined and used for the calculation of Spearman’s rank correlation coefficient to predict potential gene regulatory networks. The correlation coefficient (ρ) for each gene pair was calculated by the built-in function “cor” in R, and a threshold of ρ ≥ |0.85| was regarded as significant co-expression. Visualization of the network was created in Cytoscape 3.6.1 [70].

## 4. Conclusions

This study is the first to investigate the chromosomal location, expression profiling, and transcriptional regulation networks of *P. ginseng* OPT genes and provide a comparative genome analysis addressing the phylogeny, gene structure, and paralogs duplication history of the OPT gene family in *P. ginseng* and 11 flowering plants. Chromosomal location analyses revealed that structural variation occurred after segmental duplication, expression profiling, and transcriptional co-expression networks analyses, which indicates that both specific and pleiotropic transcription regulators might be involved in the regulation of OPT genes’ expression. Phylogenetic analyses suggested two well-supported clades in the OPT family, which can be further classified into 12 or 19 distinct groups. Motif compositions are conserved in each clade and clade-specific motifs were frequently occupied within each clade. Estimations for paralogs divergence history indicated that the majority of OPT paralogs in *P. ginseng* might have emerged from recent duplications, which was different from the history of *Arabidopsis* or cassava. The study of expression profiles in different organs and tissues of *P. ginseng* has provided insights into possible functional divergence among OPT members and important functional roles in the plant development of some OPT members. These data may provide valuable information for future functional investigations of this gene family.

## Figures and Tables

**Table 1:** Statistics of OPT genes predicted from *P. ginseng* and 11 flowering plants.

Species	Predicted	De-Redundant	Final
Ginseng	39	37	37
*Arabidopsis*	18	16	17
Rice	12	10	18
Sorghum	38	26	26
Carrot	25	16	16
Potato	42	29	29
Tomato	23	17	17
Cassava	29	21	21
Clover	31	25	25
Cacao	27	19	19
Poplar	54	28	28
Grape	26	24	23
Total	364	268	276

37 OPT genes were left after manual curation for OPT genes from Ginseng. OPT genes predicted for *Arabidopsis* and rice were replaced with 17 and 18 reviewed OPT genes retrieved from Swiss-Prot. De-redundant indicated only one gene could be kept if there were genes that had 100% similarity to it.

## Supplementary Materials

See the word file of “The list of supplementary materials.” All supplementary materials are available online.
