# Supplementary material for: Genome-Wide Identification and Comparative Analysis for OPT Family Genes in Panax ginseng and Eleven Flowering Plants
Source: Molecules. 2018 Dec 20;24(1):15. doi: 10.3390/molecules24010015 (PMC6337337; doi:10.3390/molecules24010015)

Supplemenatry file 3: Sequence logo of different motifs identified in the OPT gene family

Motif 1


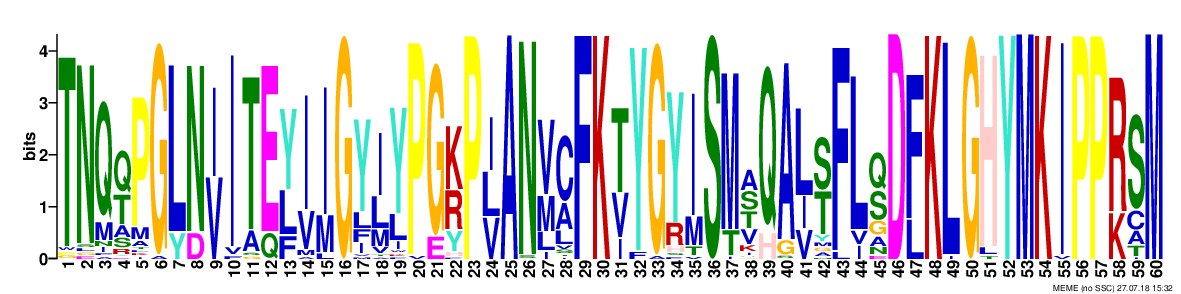


Motif 2


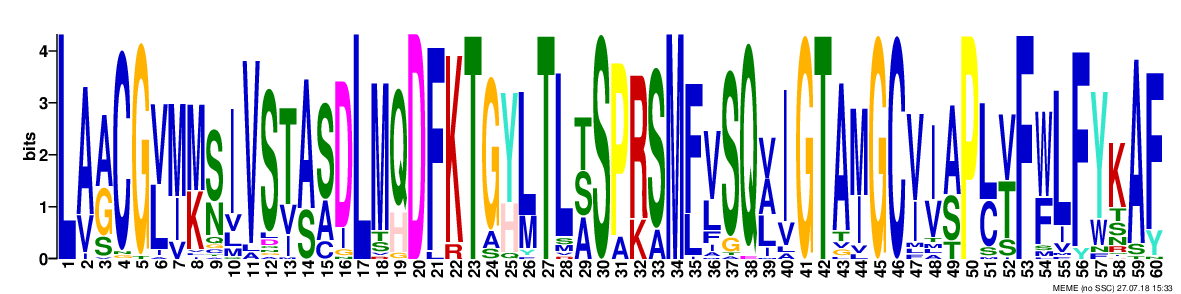


Motif 3


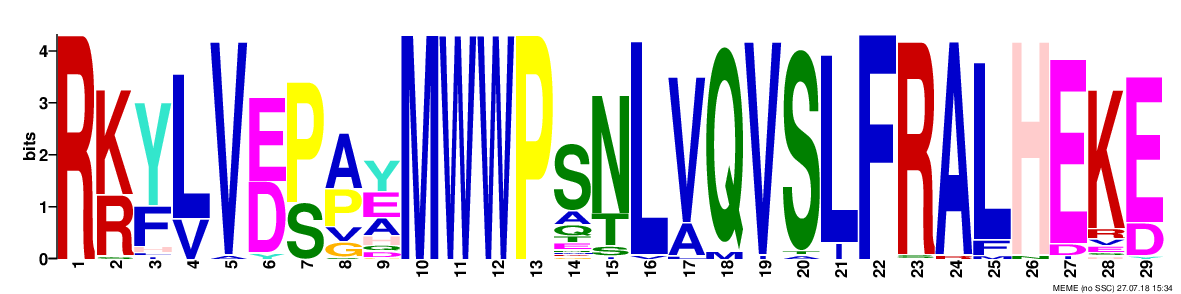


Motif 4


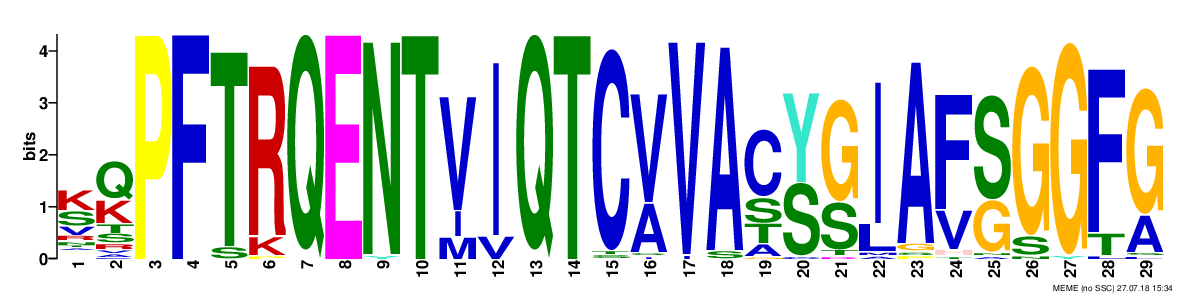


Motif 5


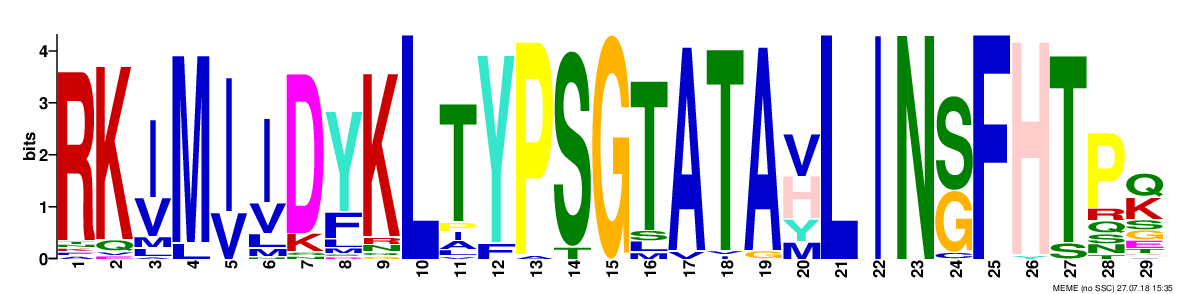


Motif 6


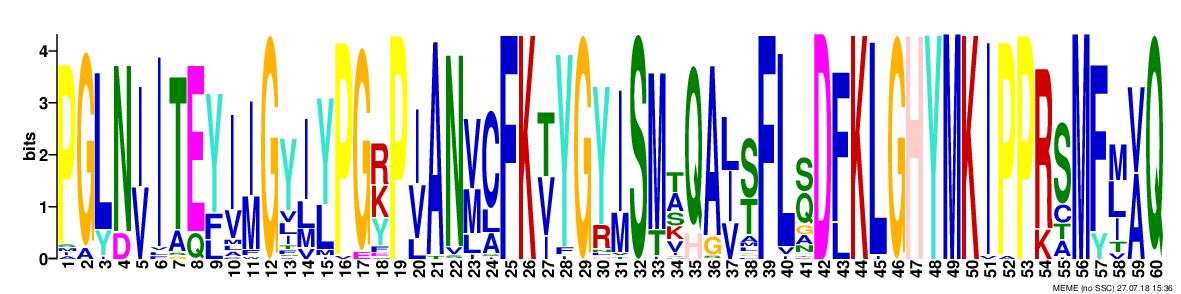


Motif 7


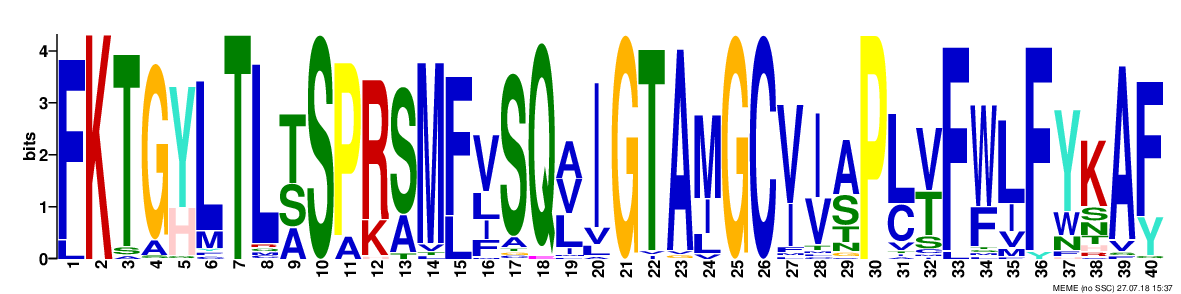


Motif 8


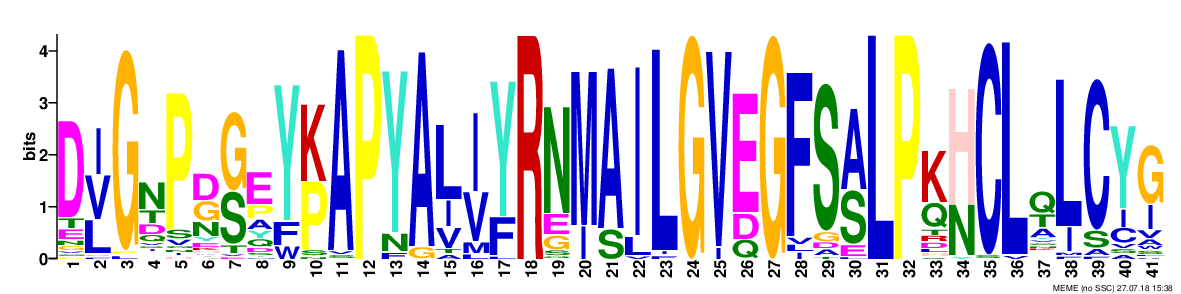


Motif 9


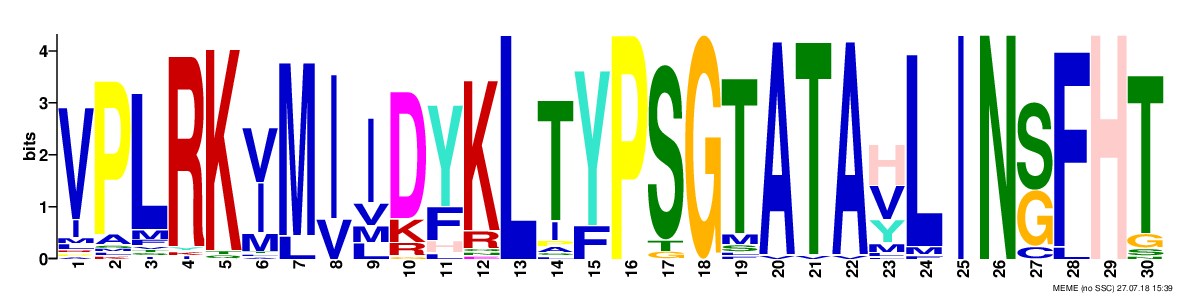


Motif 10


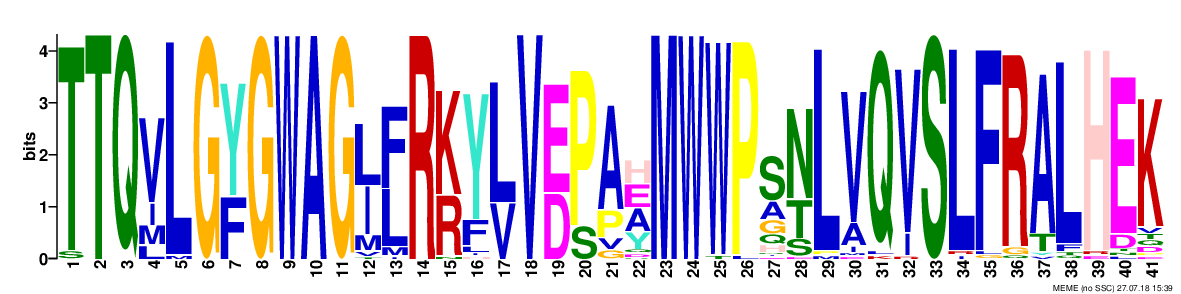


Motif 11


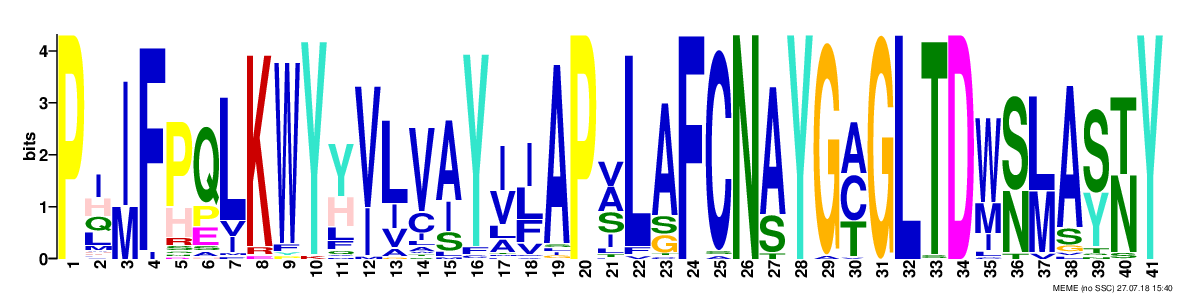


Motif 12


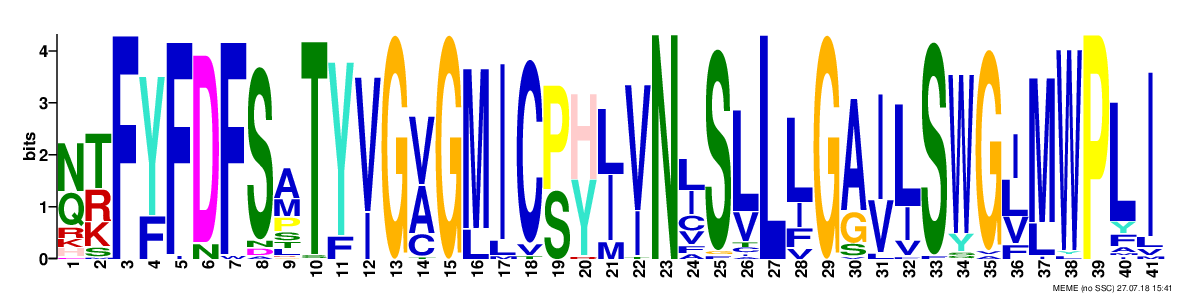


Motif 13


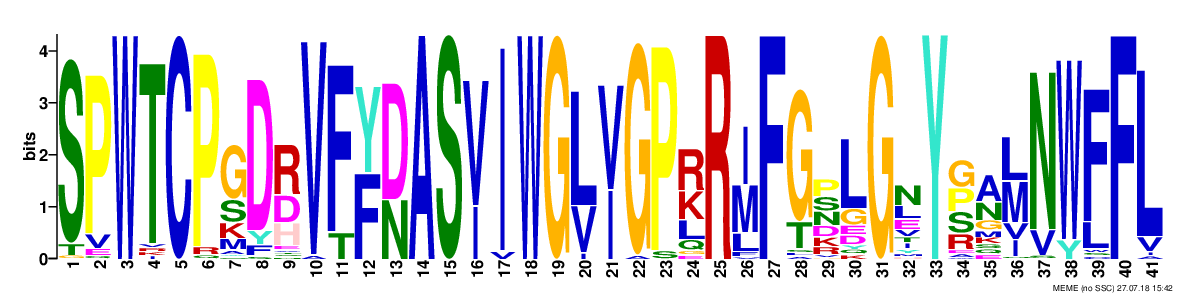


Motif 14


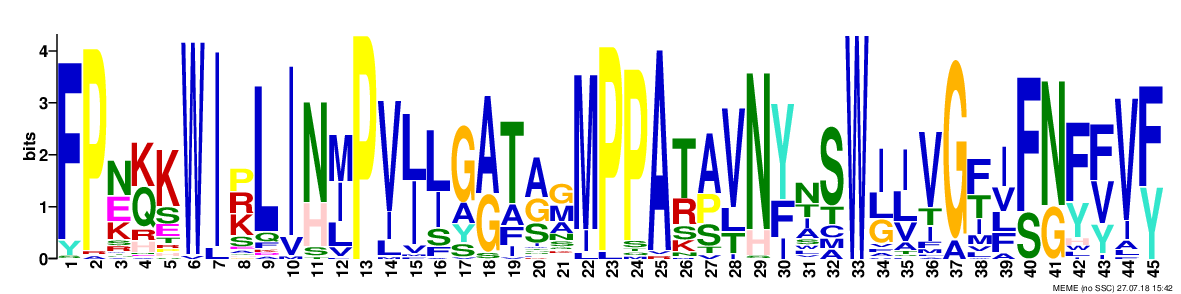


Motif 15


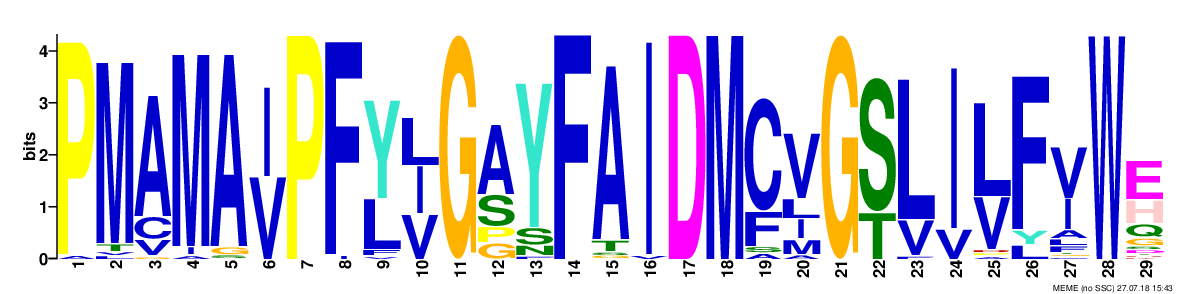


Motif 16


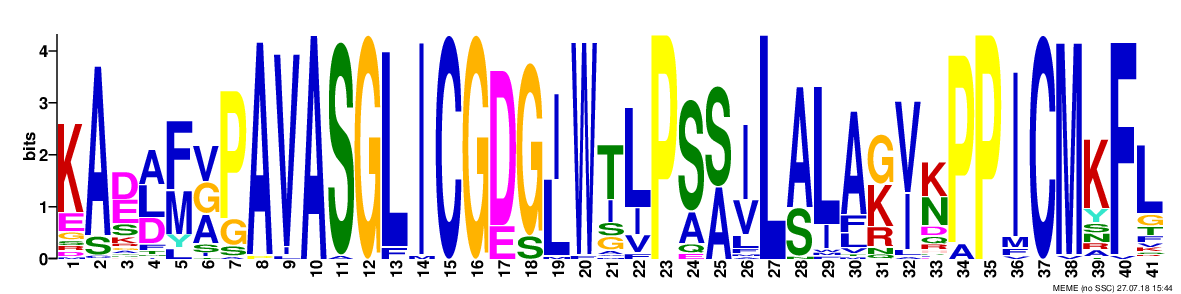


Motif 17


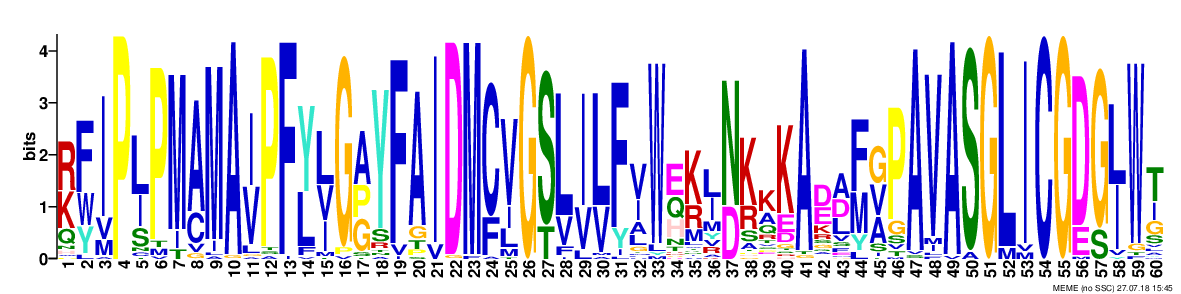


Motif 18


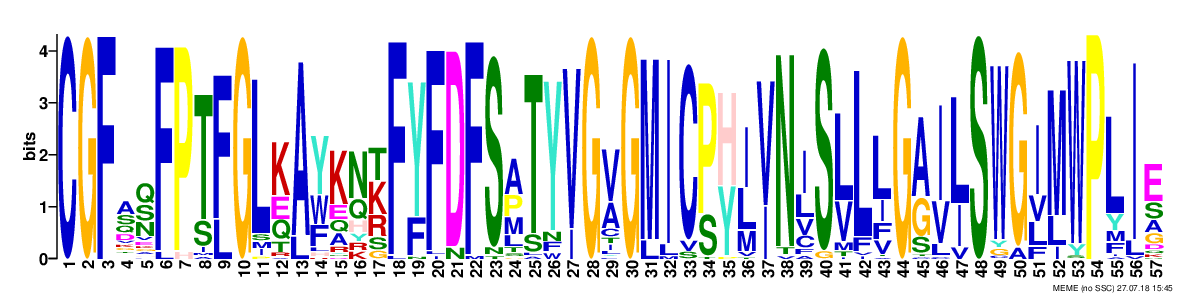


Motif 19


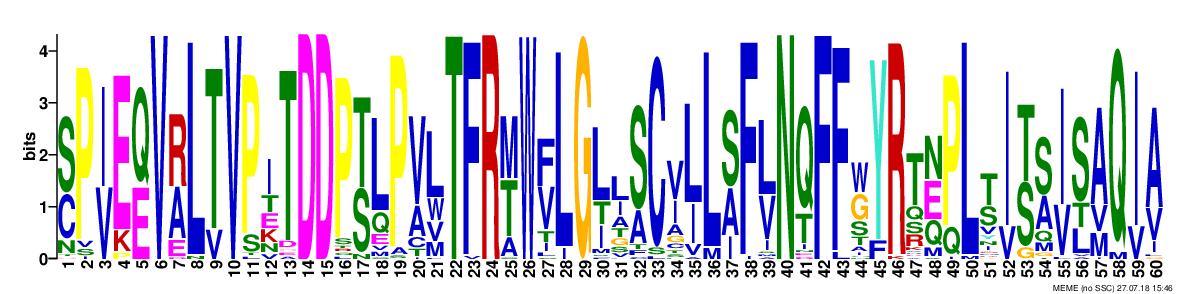


Motif 20


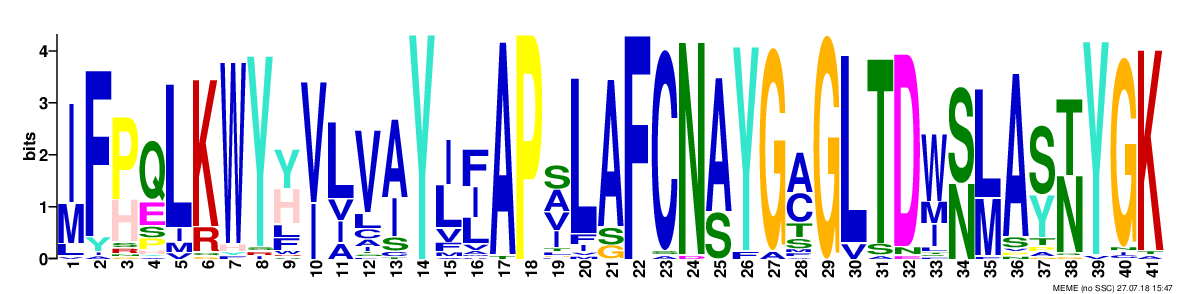


Motif 21


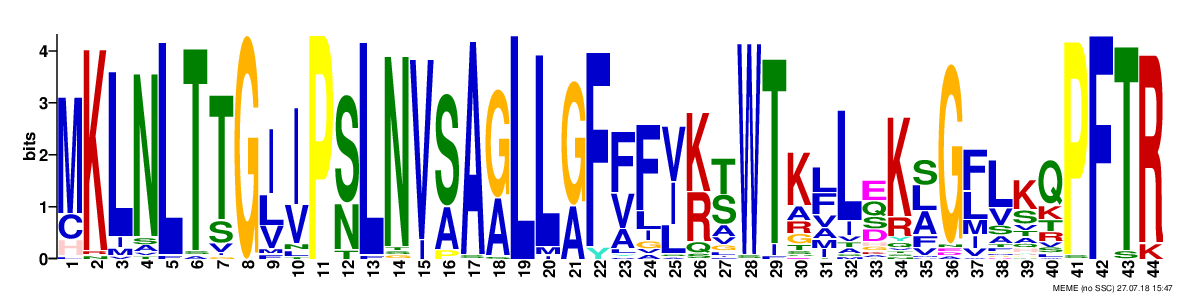


Motif 22


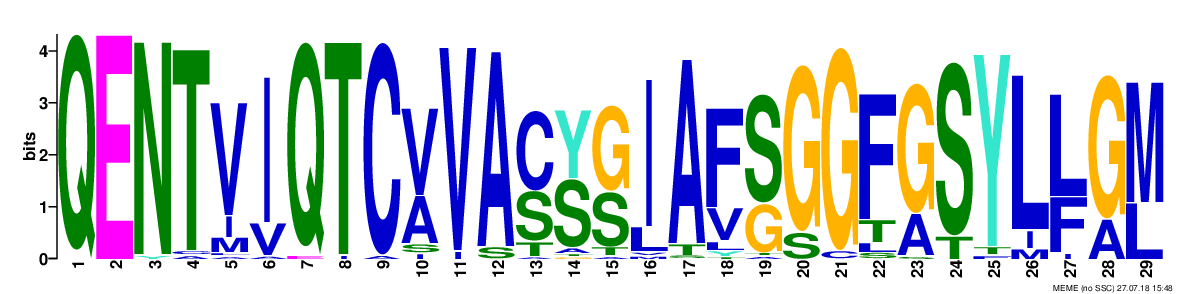


Motif 23


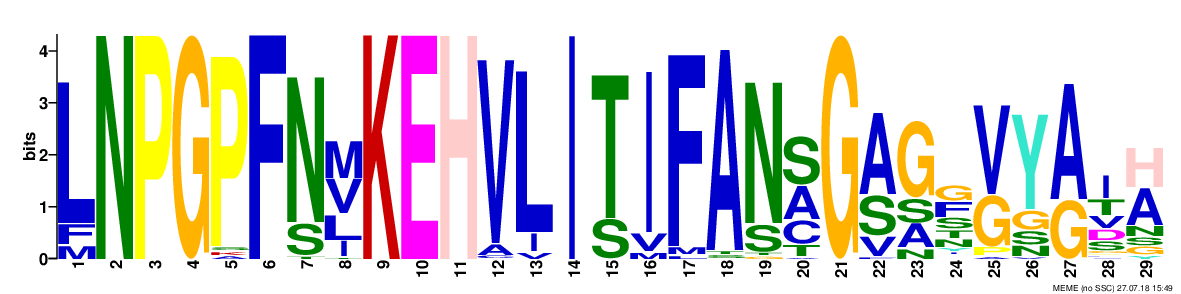


Motif 24


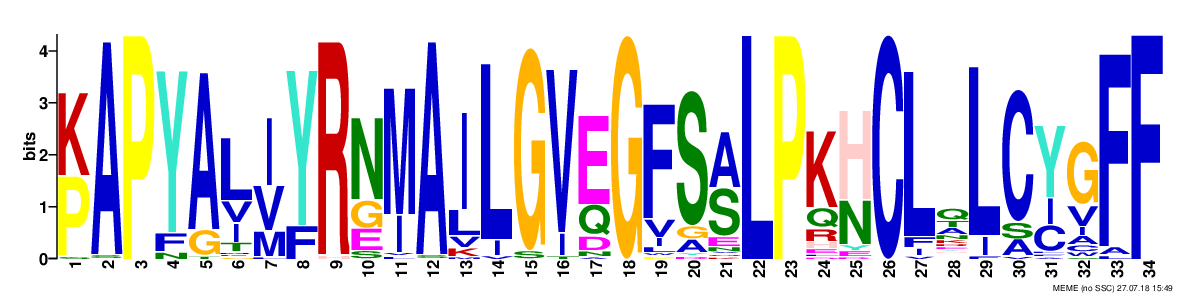


Motif 25


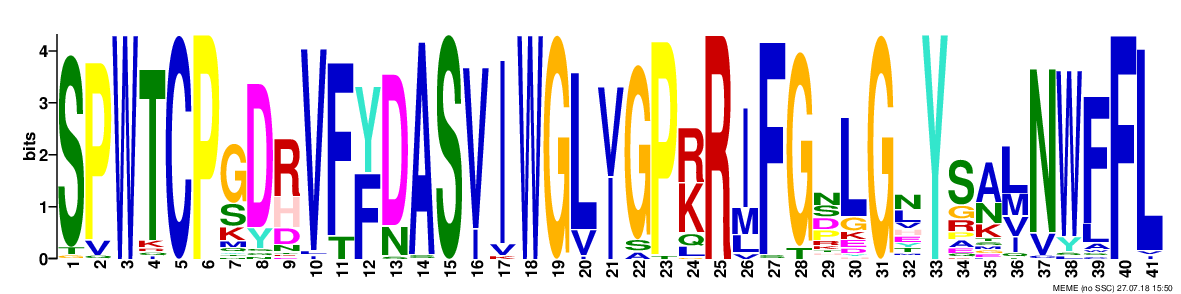


Motif 26


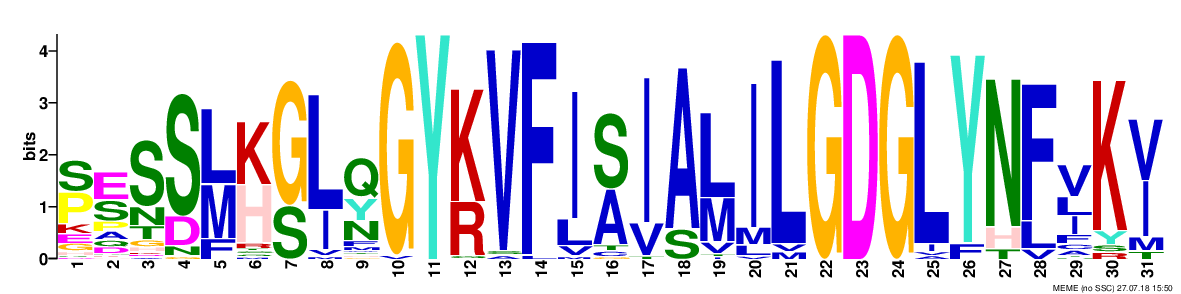


Motif 27


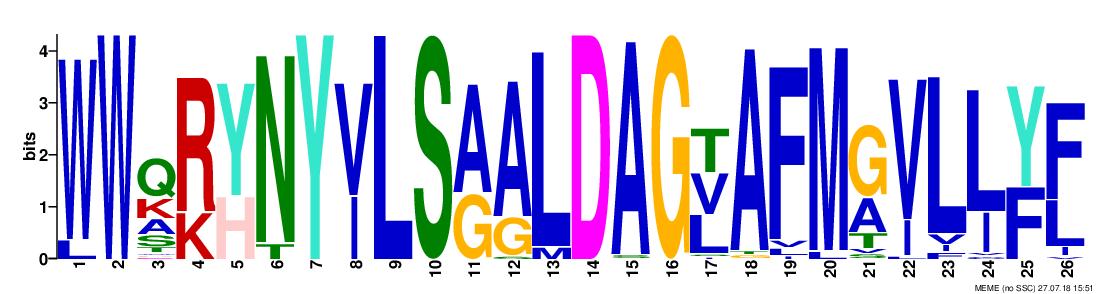


Motif 28


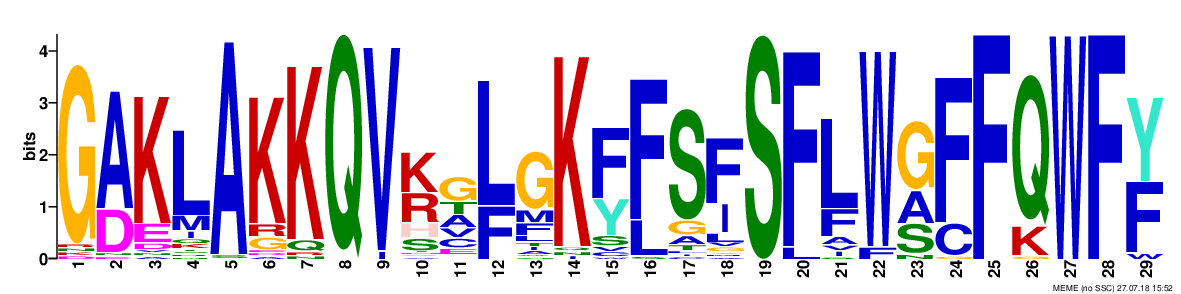


Motif 29


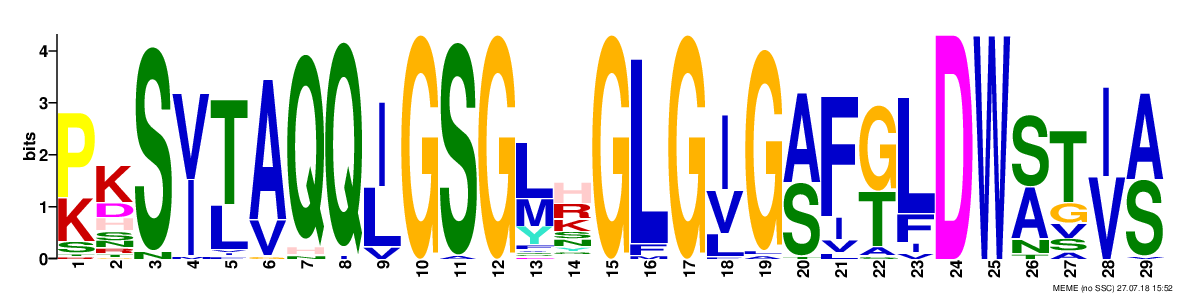


Motif 30


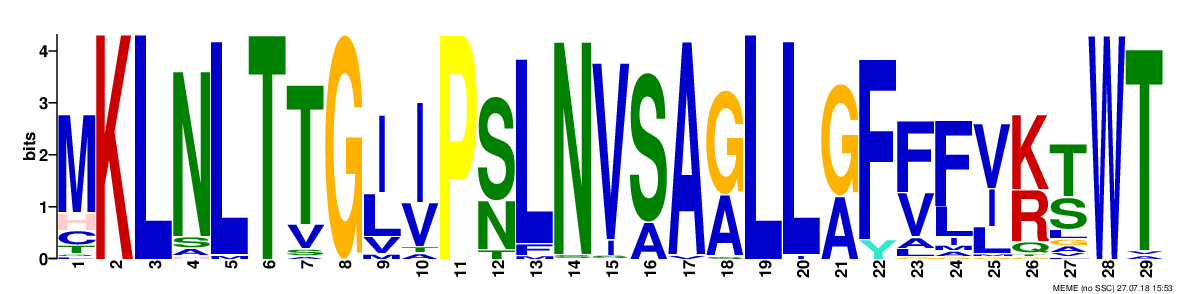

Supplement: Supplementary file 1 [file molecules-24-00015-s001.zip › Supplementary files/Supplementary material 5-Supplementary file 3.docx]
