# Supplementary material for: Genome-Wide Identification and Comparative Analysis for OPT Family Genes in Panax ginseng and Eleven Flowering Plants
Source: Molecules. 2018 Dec 20;24(1):15. doi: 10.3390/molecules24010015 (PMC6337337; doi:10.3390/molecules24010015)
Supplement: Supplementary file 1 [file molecules-24-00015-s001.zip › Supplementary files/The list of supplementary materials.docx]

**The list of supplemntary materials:**

**Supplementary Material 1: Supplementary file 1:** proteins sequences of 278 OPT genes from *Panax ginseng* and 11 flowering plants

**Supplementary Material 2: Table S1-S8. Table S1**, Predictions for protein properties and membrane locations for OPT genes from *Panax ginseng* and 11 flowering plants; **Table S2,** Candidate OPT paralogs identified from *Panax ginseng* and 11 flowering plants; Table S3, Location-related OPT genes for *Panax ginseng*; **Table S4,** Statistics for gene numbers in PT and YSL clade shared by each motif; **Table S5**, Matrix of correlation coefficient between OPT genes and transcription regulators calculated from expression profiles of *Panax ginseng* root; **Table S6,** Strong links between transcription factors and OPT genes from *Panax ginseng* root; **Table S7,** Number of candidate transcrition regulators co-expressed with OPT genes in *Panax ginseng* root; **Table S8,** Number of candidate OPT genes co-expressed wth transcription regulators in *Panax ginseng* root

**Supplementary Material 3: Figure S1.** Motif compositions for OPT genes identified from *Panax ginseng* and 11 flowering plants

**Supplementary Material 4: Supplementary file 2.** Prediction results of conserved domains for OPT gene family

**Supplementary Material 5: Supplemenatry file 3.** Sequence logo of different motifs identified in the OPT gene family

**Supplementary Material 6: Supplementary file 4.** Prediction results of transcription factors for *Panax ginseng* using PlantTFcat

**Supplementary Material 7: Supplementary file** **5.** XML output of motif analysis by MEME software for all OPT genes.
