# Supplementary material for: Genome-Wide Identification and Comparative Analysis for OPT Family Genes in Panax ginseng and Eleven Flowering Plants
Source: Molecules. 2018 Dec 20;24(1):15. doi: 10.3390/molecules24010015 (PMC6337337; doi:10.3390/molecules24010015)
Supplement: Supplementary file 1 [file Supplementary material 3-Figure S1.pdf › Supplementary files/Supplementary material 3-Figure S1.pdf]

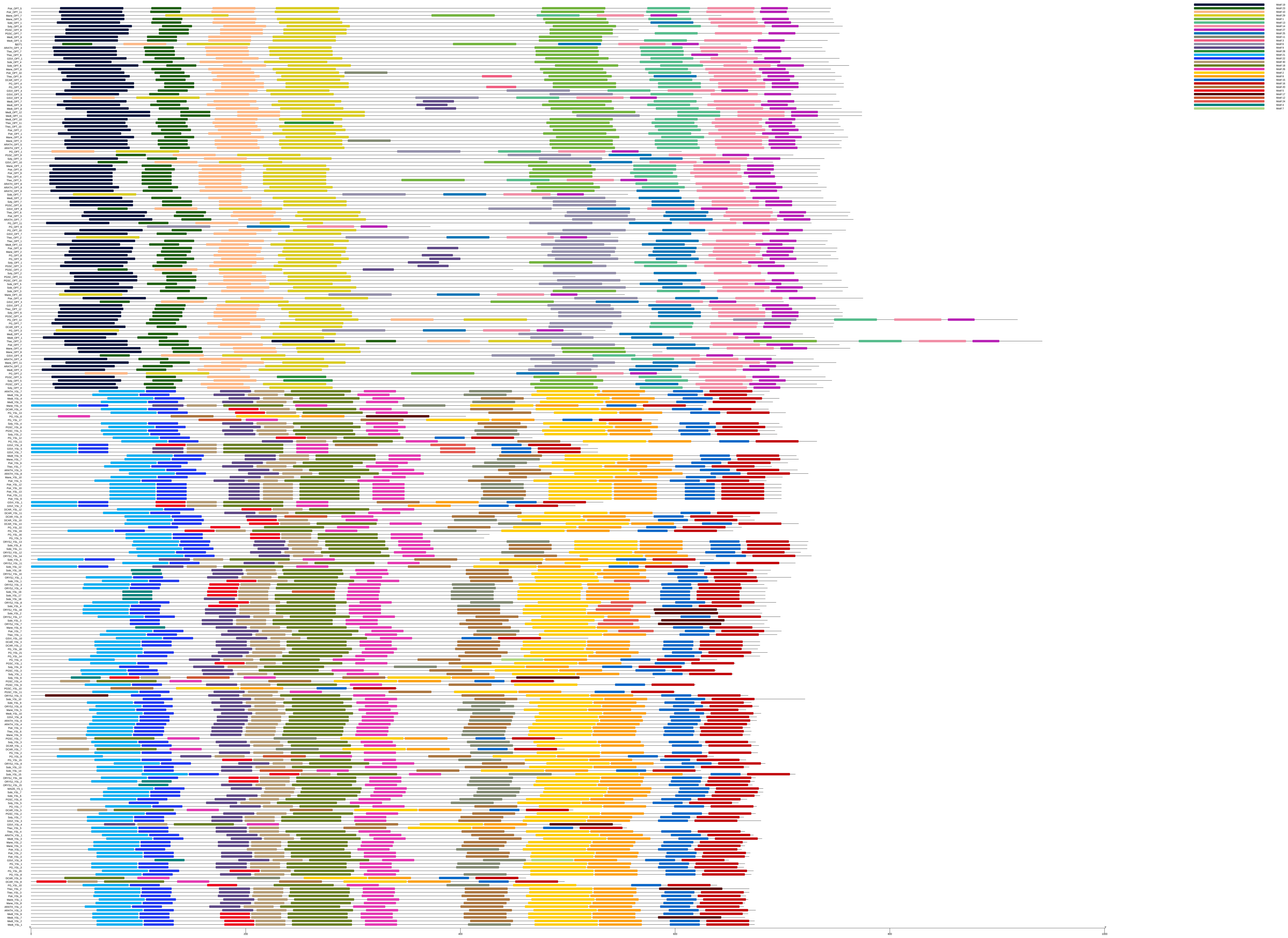

0
200
400
600
800
1000
Motif 19
Motif 23
Motif 10
Motif 29
Motif 1
Motif 13
Motif 14
Motif 27
Motif 25
Motif 11
Motif 3
Motif 6
Motif 9
Motif 28
Motif 21
Motif 22
Motif 30
Motif 18
Motif 26
Motif 2
Motif 8
Motif 15
Motif 16
Motif 20
Motif 5
Motif 17
Motif 12
Motif 24
Motif 4
Motif 7
